# Relationship Between Big Five Personality and Pathological Internet Use: Mediating Effects of Loneliness and Depression

**DOI:** 10.3389/fpsyg.2021.739981

**Published:** 2021-12-16

**Authors:** Yong Zhou, Hui Li, Lei Han, Suyue Yin

**Affiliations:** Shandong Normal University, Jinan, China

**Keywords:** Big Five personality, pathological Internet use, loneliness, depression, mediating effects

## Abstract

Pathological Internet use will have a serious impact on normal individual study and work and has become one of the most important factors hindering the growth and development of contemporary college students. The purpose of this study was to examine the mechanisms of loneliness and depression in the relationship between the Big Five personality traits and pathological Internet use (PIU). A total of 1,179 college students were studied using the Chinese version of the Big Five Personality Scale, Loneliness Scale, Depression Scale, and Pathological Internet Use Scale. The results showed that loneliness partially mediated the relationship between extraversion and PIU and completely mediated the relationship between agreeableness and PIU and neuroticism and PIU. Depression completely mediated the relationship between agreeableness and PIU, neuroticism completely mediated the relationship with PIU, and sense of responsibility partially mediated the relationship with PIU. This study revealed the relationship between different personality traits and PIU and the mediating role of loneliness and depression, suggesting that we should carry out targeted interventions on PIU for college students with different personality traits.

## Introduction

The annual usage of demand for the Internet is growing rapidly. The 47th Statistical report on the development of the Internet in China shows that by December 2020, the scale of China’s Internet users had reached 989 million, among which college students were one of the main groups of Internet users. For college students, in the era of “Internet +,” computers, mobile phones, and other network media can be found in every corner of the campus, making the network an emerging platform for the dissemination of knowledge and information. Research shows the rapid growth trend of college student users, who have become the main participants of networks (Zsila et al., 2018). The popularization and development of the Internet have brought unprecedented benefits to human life. For example, people communicate with each other more closely and conveniently, and the distance between people has shortened. However, increasingly easier access to information from the outside world also brings some adverse effects (Billieux et al., 2015; Schimmenti et al., 2021). For example, previous studies have pointed out that excessive Internet use may lead to pathological Internet use (PIU; Zhang et al., 2018).

Pathological internet use refers to behaviors that cause significant damage to the social and psychological functions of individuals from the excessive use of the Internet, seriously affecting one’s normal learning and work (Musetti and Corsano, 2018; Zhou et al., 2018). PIU is a mental disorder involving pathological behavior and cognitive maladjustment associated with Internet use. PIU, similar to pathological gambling, alcohol dependence, and drug addiction, is an addictive behavior that damages individuals’ physical and mental health, learning, interpersonal health, and life to varying degrees (Hou et al., 2021). PIU has become one of the most important factors hindering contemporary college students from experiencing healthy growth (Musetti et al., 2018). PIU also has a certain negative impact on the cultivation of correct values and the ideological quality of contemporary college students (Jiao, 2017). In addition, several previous studies have pointed out that PIU has a negative impact on the mental health of individuals, such as reducing their vitality and happiness (Akın, 2012), and leading to loneliness, depression, and other negative emotional experiences (Ayas and Horzum, 2013; Musetti et al., 2020). We reviewed the relevant literature on pathological Internet use and found similar results. A study of young people in India, Mexico, the Philippines, and Turkey found that several factors underlie the growing problematic Internet use in young people. These factors include emotional distress, a need for escapism, loneliness, and social media use (Fernandes et al., 2021). A study of undergraduate students at the Bhilai Institute of Technology, Durg and Yugantar Institute of Technology and Management, Rajnandgaon, Chhattisgarh, shows that students suffering from stress, anxiety, and depression may tend to excessively use the Internet to relieve low mood, insomnia, fearfulness, feelings of guilt, and hopelessness (Ranjan et al., 2021). Therefore, it is important to investigate the antecedent variables that affect PIU.

Studies have found that the PIU of individuals is related to Big Five personality traits (Musetti et al., 2016; Stodt et al., 2018). A recent meta-analysis demonstrated a significant positive correlation between neuroticism and PIU. Highly neurotic teenagers tend to be overly sensitive to interpersonal relationships (Kong et al., 2011) and are prone to social problems in real life (Chinneck et al., 2018). However, Internet socialization has the characteristics of nonface-to-face, anonymity, and immediacy, which can alleviate social problems to a certain extent (Yang et al., 2019). The social compensation model points out that when individuals gain social satisfaction on the Internet, they tend to use more social networks (Gao et al., 2018). Agreeableness, extraversion, conscientiousness, and openness are negatively correlated with PIU (Wang and Zhao, 2000). Conscientiousness type students show the characteristics of being organized, enterprising, and self-disciplined. They can control their online behavior well and rarely cause PIU (Zhang and Zhang, 2021). Individuals with agreeableness and extraverted personality types are friendly, sincere, cheerful, and altruistic. Students of this type have good interpersonal relationships and do not easily indulge on the Internet. Individuals with open personality types are shown to love fantasy and have rich emotions and keen insights. In the process of using the Internet, they can also reasonably use it for entertainment, learning, and other activities (Chen et al., 2011). However, previous studies focused on the relationship between the Big Five personality traits and PIU but ignored the internal mechanisms. Therefore, it is also very important to explore the mechanism of the Big Five personality traits on PIU and to provide suggestions for the prevention and intervention of PIU by individuals with different personality types.

### The Mediating Role of Loneliness

Williams (1983) defined loneliness as a painful experience when an individual subjectively feels a lack of or dissatisfaction with interpersonal relationships. The inner feeling of being ignored, omitted, or disregarded by others is a negative feeling with respect to emotion and cognition (Yang et al., 2005). Some personality defects are also an important factor causing loneliness. Individuals with different levels of loneliness have different personality characteristics (Heinrich and Gullone, 2006). People with agreeableness and extraverted personalities are less likely to feel lonely because they usually have a good interpersonal network and more social support. Individuals with strong neurotic personalities are likely to be emotional and impulsive and are more dependent. They easily escape reality and are prone to interpersonal problems in real life. Therefore, they may lack social support and experience more loneliness (Huang et al., 2013). College students with a strong sense of responsibility are more restrained and rigorous and have stronger organization skills, tendency to plan actions, and self-control. However, given a lack of social experience, they are full of hope and fear for the future. The feeling of being understood by others can cause loneliness, but it is not very significant (Zhang et al., 2007). Individuals with strong openness are curious and good at accepting and applying new concepts.

Previous studies have found that lonely individuals are more likely to develop PIU, and loneliness—as a negative emotional experience—will affect individual learning and life. To alleviate the negative experience, lonely individuals may play games to relieve their mood (Chen et al., 2011); however, spending too much time on online games fosters more PIU (Amichai-Hamburger and Ben-Artzi, 2003). In addition, college students with a strong sense of loneliness are overly sensitive to interpersonal relationships because of the lack of effective social interaction. In this case, they avoid social and interpersonal relationships and use the Internet and cyberspace as a substitute for social interaction. The social compensation model points out that when individuals find that online social interaction can meet their needs and bring a pleasant social experience, they may invest more energy in online social interaction that may eventually lead to PIU (Choi et al., 2018). Therefore, these factors basically have no effect on loneliness.

### The Mediating Role of Depression

In addition, studies have found that depressed individuals also face a high risk of PIU (Long et al., 2021). Depression is a relatively common affective disorder that hinders individual psychological adjustment (Xiao et al., 2006). The current standard used in the diagnosis of depression in China is the World Health Organization standard, also called ICD10, which is the tenth edition of the International Classification of Diseases and Diagnostic Standards. The diagnostic criteria for depression are based on the fact that the depressive episode must last for two weeks without a manic episode, and it is not a mental disorder caused by psychoactive substances and other organic disorders. PIU mainly includes two major types: depressive episodes, that is, first depressive episodes, first-onset depression, and recurrent depression. There are three core symptoms of depressive episodes. The first is depressive mood, which is sadness and unhappiness; the second is no interest or pleasure in activities that are usually of interest; and the third is a lack of energy. These are the three core symptoms. There are seven additional symptoms, such as lack of self-confidence, self-blame, suicidal thoughts, indecision, inattention, changes in mental activity, sleep disorders, and changes in appetite.

Hu and Li (2010) believed that the degree of PIU was positively correlated with the degree of depression; that is, a more severe depression resulted in a deeper degree of PIU (Hu and Li, 2010). College students with depressed moods are depressed, negative, have less communication with their peers, and have a poor sense of collective identity. They are more likely to use the Internet to relieve bad emotions and escape reality. Actually, they treat the Internet as a “drug.” Therefore, such individuals with psychological problems will rely on the Internet, which in turn leads to PIU (Feng et al., 2007). Research also exists that suggests the people with depression have increased brain dopamine levels after surfing the Internet for a long time, which can make them highly excited for a short period; however, subsequently, their depression will be more severe than before (He et al., 2012) Over time, a vicious circle develops; that is, when the degree of PIU deepens, people become increasingly inseparable from the Internet, which will produce stronger depression and further deepen the degree of PIU (Zhang et al., 2009).

Studies have found that neuroticism has the most significant impact on depressive emotions (Bagby et al., 1995). Neuroticism is believed to possibly be a susceptibility factor for major depression. College students with a high degree of neuroticism tend to be emotionally unstable and have a weaker ability to adapt to the environment, which likely causes depression (Zhang et al., 2015).

Although college students with a strong sense of responsibility have strong planning skills because of their higher requirements and lack of practical experience, they are prone to encounter setbacks in the process of engaging in activities, which can trigger depression and have a significant impact on depression. College students with agreeableness characteristics are more generous, believe that human nature is inherently good, and—because they value cooperation and interpersonal harmony—are not prone to depression (Zhao et al., 2010). People with extraversion and an open personality, such as being in contact with others, are full of energy, can often experience positive emotions, and have no significant relationship with depression (Xia et al., 2014). Because the Big Five is related to depression, depression is significantly related to PIU, and a Big Five personality trait may also affect individual PIU through the mediating effect of depression.

### This Study

The research question of this study is what is the relationship between different personality traits and pathological Internet use? What role do loneliness and depression play in the interaction between personality traits and pathological Internet behavior? Given these questions, the following assumptions are made in this study:

*H1*: Different personality traits have significant predictive effects on PIU.

*H2*: Different personality traits can indirectly predict PIU through the mediating effect of loneliness.

*H3*: Different personality traits can indirectly predict PIU through the mediating effect of depression.

## Materials and Methods

### Participants

A cluster sampling method was adopted in 2019 to select 40 classes from 3 grades in a university in Shandong Province from different majors, and paper and pencil tests were carried out in class. A total of 1,200 questionnaires were distributed, and 1,188 questionnaires were recovered, for a recovery rate of 99%. Of the returned questionnaires, unserious answers and missing values were excluded, and so 61 participants’ data were excluded in the final analysis (specifically, 22 participants chose the same option for the answer to more than ten subjects, 18 participants filled out incomplete questionnaires, and 21 participants left missing values for more than ten subjects in the questionnaire). Finally, 1,127 valid questionnaires were obtained for an effective rate of 93.92%. The data included in the final analysis were from 687 male students (58%) and 440 female students (42%), with 531 in science and engineering and 596 in the liberal arts. The ages ranged from 16 to 24 years, with an average of 19.8 ± 1.9 years. The time range of the students involved in the test was 1 to 12 years, with an average of 7.3 ± 2.3 years. In addition, the study was approved by the local ethics committee, and prior consent was obtained from the testing school and students. All of the students who participated in the questionnaire voluntarily signed an informed consent, and the questionnaire was conducted anonymously.

### Measuring Tools

#### Big Five Personality

This study uses the Chinese version of the Big Five Personality Scale (Wang et al., 2011a), which includes 44 questions and consists of five dimensions: extraversion, agreeableness, neuroticism, responsibility, and openness. The extroversion dimension has four reverse scoring titles, the agreeableness dimension has eight reverse scoring titles, the neuroticism dimension has four reverse scoring titles, the sense of responsibility dimension has four reverse scoring titles, and the openness dimension has 5 reverse scoring titles. Using the original points scoring method, the relevant dimensions of personality became more obvious as the score increased. For example, “may be moody,” “be considerate of others, and be kind to almost everyone.” A 5-point scale is used (1 = strongly disagree and 5 = strongly agree). The internal consistency coefficient in this study is 0.81. In addition, confirmatory factor analysis shows that the scale has good construct validity: *χ*^2^(167) = 426, CFI = 0.96, NFI = 0.94, NNFI = 0.97, RMSEA = 0.07, AVE = 0.30, and CR = 0.95.

There was significant correlation between all variables (*p* < 0 0.01), the absolute values of correlation coefficients are all less than 0.5, and are all less than the square root of AVE, which indicates that each latent variable has a certain correlation with each other and a certain degree of differentiation between them, which indicates that the discriminant validity of the scale data is ideal (Table 1).

**Figure 1:**
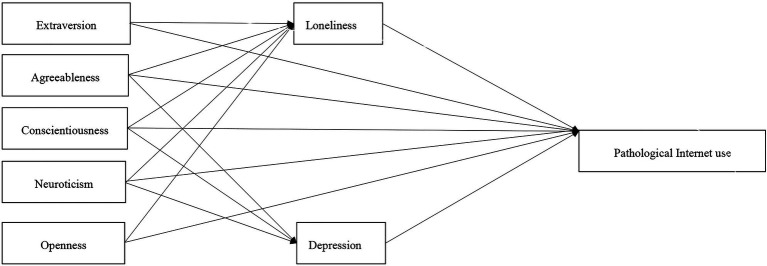
Hypothetical model.

**Table 1:** Discriminant validity analysis.

	Extraversion	Agreeableness	Conscientiousness	Neuroticism	Openness
Extraversion	0.233				
Agreeableness	0.145^***^	0.209			
Conscientiousness	0.116^***^	0.051^***^	0.194		
Neuroticism	−0.196^***^	−0.074^***^	−0.057^***^	0.174	
Openness	0.220^***^	0.072^***^	0.077^***^	−0.063^***^	0.232
The square root of AVE	0.483	0.458	0.440	0.417	0.482

*** *p < 0.01, the diagonal is the extraction of AVE evaluation variance variation*.

#### Loneliness

This study uses the Sinicized Modified Loneliness Scale (Wu et al., 2010), a single-dimension scale with 20 items, of which 9 are scored in reverse. The degree of loneliness increases with the score, such as “Do you often feel harmonious with the people around you?” and “Do you often feel the lack of partners?” The scale uses 4-point scoring (1 = strongly disagree and 4 = strongly agree). The internal consistency coefficient in this study is 0.84. In addition, confirmatory factor analysis showed that the scale has good structural validity: *χ^2^*(132) = 401, CFI = 0.97, NFI = 0.97, NNFI = 0.97, RMSEA = 0.05, AVE = 0.41, and CR = 0.93.

#### Depression

This study uses the Sinicized Modified Depression Scale (Wang et al., 2011b), a single-dimension scale with 20 items, of which 4 are scored in reverse. The degree of depression increases with the score, such as “I feel that even with the help of family or friends, I cannot get rid of depression,” and “I am not disturbed by those who usually My things are bothering.” The scale uses 4-point scoring (1 = strongly disagree and 4 = strongly agree). The internal consistency coefficient in this study is 0.84. In addition, confirmatory factor analysis shows that the scale has good construct validity: *χ^2^*(132) = 368, CFI = 0.97, NFI = 0.97, NNFI = 0.97, RMSEA = 0.05, AVE = 0.42, and CR = 0.93.

#### Pathological Internet Use

This study uses the Chinese revised PIU scale (Lei and Yang, 2007), a single-dimension scale with 11 items,. No questions need to be scored backward. The degree of pathological Internet use increases with the score, such as “No matter how tired, I always feel energetic when surfing the Internet,” and “the Internet has a negative impact on my health,” using 7-point scoring (1 = strongly disagree and 7 = strongly agree). The internal consistency coefficient in this study is 0.90. In addition, confirmatory factor analysis shows that the scale has good structural validity: *χ^2^*(124) = 316, CFI = 0.98, NFI = 0.97, NNFI = 0.97, RMSEA = 0.07, AVE = 0.55, and CR = 0.93.

### Procedures and Data Processing

The tests are organized by taking one class as a unit. Unified written instructions for collective testing were used. The authenticity of the answers and the confidentiality of the results in the instructions were emphasized. SPSS 23.0 and AMOS 24.0 statistical software were used for a statistical analysis of the data.

We first used SPSS 23.0 for data collation and conducted internal consistency reliability analysis and convergent validity analysis of the questionnaires, followed by descriptive statistics and correlation analysis. AMOS 24.0 was used to test the structural validity, discriminant validity, and combinatorial reliability of the questionnaire. and then, the hypothesis model was tested. For the reliability and validity analysis of the measurement questionnaire, it is generally required that the internal consistency reliability coefficient is higher than 0.8, *χ^2^/df* is lower than 5, CFI, NFI, and NNFI are higher than 0.9, RMSEA is lower than 0.08, AVE is higher than 0.5, and CR is higher than 0.7.

## Results

### Common Method Deviation Test

Because all variables in this study are used in the questionnaire survey method—all derived from self-reports by the subjects—common method deviations may exist. Therefore, when we analyzed the data, we tested the common method deviation of the three variables involved in the study, that is, the Big Five personality traits, loneliness, and depression. This study adopted Harman’s single-factor test, which can be used in the questionnaire study. Exploratory factor analysis, which many researchers have used, is performed on each topic. In all projects, 55 factors had initial eigenvalues greater than 1, and the largest factor explained only 11.13% of the variation, which was much less than the critical standard of 40%. Therefore, no serious common method bias problem exists in this research.

### Correlation Analysis of Big Five Personality Traits, Loneliness, Depression, and PIU

Correlation analysis was conducted using the total scores of the Big Five personality traits, loneliness, depression, and PIU. All variables were significantly correlated (Table 2). Extraversion, agreeableness, a sense of responsibility, and openness were negatively correlated with loneliness, depression, and PIU (ps < 0.01); neuroticism was positively correlated with loneliness, depression, and PIU (ps < 0.01); and loneliness and depression were positively correlated with PIU (ps < 0.01).

**Table 2:** Describes the statistics and correlation analysis.

Variables	*M ± SD*	1	2	3	4	5	6	7	8
1. Extraversion	25.52 ± 4.65	1							
2. Agreeableness	32.22 ± 4.85	0.42^**^	1						
3.Conscientiousness	29.01 ± 4.71	0.33^**^	0.35^**^	1					
4. Neuroticism	20.20 ± 3.84	−0.51^**^	−0.41^**^	−0.41^**^	1				
5. Openness	33.07 ± 4.81	0.39^**^	0.31^**^	0.33^**^	−0.21^**^	1			
6. Loneliness	41.42 ± 8.59	−0.37^**^	−0.43^**^	−0.23^**^	0.44^**^	−0.19^**^	1		
7. Depression	17.01 ± 9.72	−0.31^**^	−0.39^**^	−0.30^**^	0.47^**^	−0.18^**^	0.64^**^	1	
8.PIU	39.43 ± 11.76	−0.14^**^	−0.19^**^	−0.36^**^	0.27^**^	−0.13^**^	0.31^**^	0.37^**^	1

** *p < 0.01*.

### SEM Analysis of Big Five Personality, Loneliness, Depression, and PIU

The results of the correlation analysis show that all of the variables involved in this study have significant correlations, meeting the prerequisites of the mediating effect test.

Based on the previous literature review, we constructed a theoretical model of Big Five personality traits to PIU. By analyzing the path coefficient, we found that the paths of conscientiousness and openness to loneliness, extraversion and openness to depression, agreeableness, neuroticism, and openness to pathologic Internet use did not reach significant levels.

**Figure 2:**
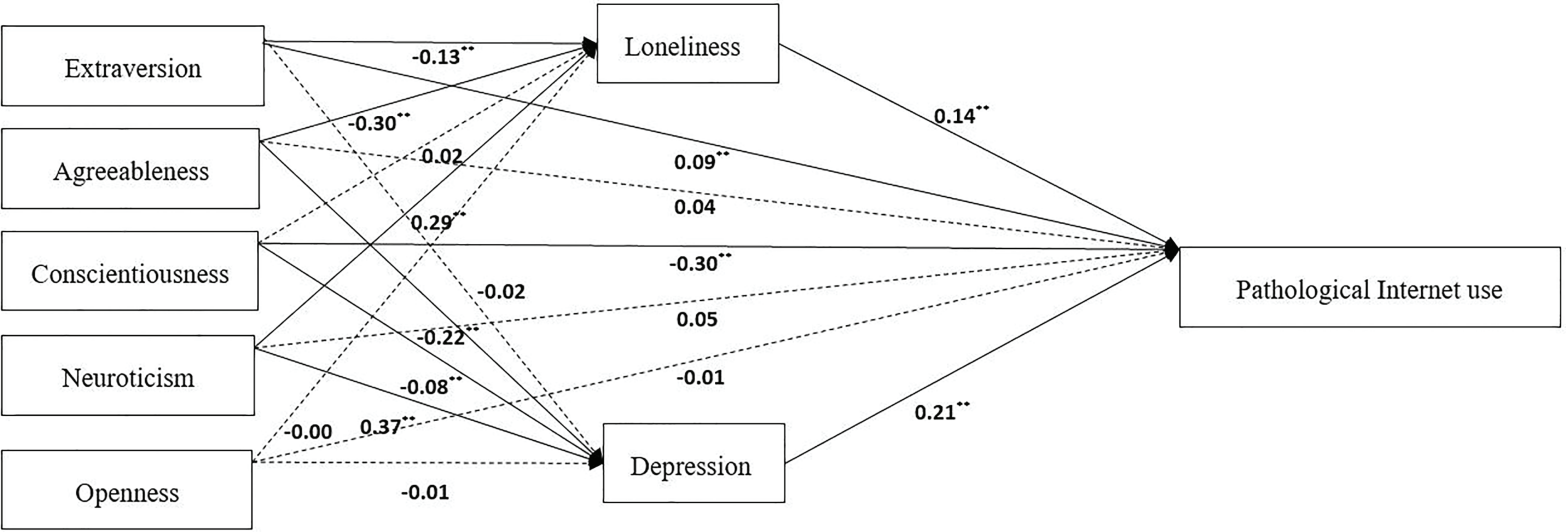
Theoretical model. ^**^p < 0.01.

**Figure 3:**
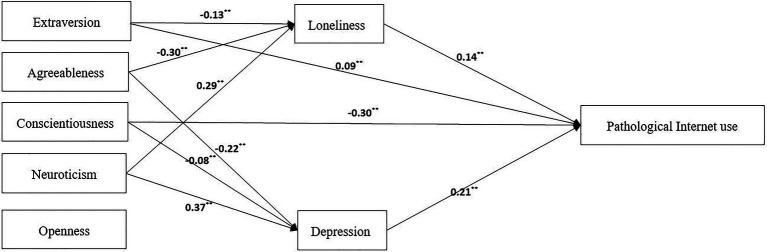
Comparison model. ^**^p < 0.01.

Therefore, these seven pathways were deleted, and a comparison model was established to determine the mechanism of the Big Five personality traits on PIU by comparing the two models (Figures 2, 3).

The comparison results of the two models show that their fitting indices have reached an acceptable level (Table 3). Because *∆c^2^* = 2.11, *∆df* = 4, and *∆c2/∆df* = 0.53 < 9.48, there is no significant difference between the two models (Wen-Zhonglin and Jietai, 2006), but the comparative model uses fewer paths to explain the mechanism of the Big Five personality traits on pathology. Because ∆R^2^ comparison = 0.77 > *∆R^2^*_theory_ = 0.70, the comparative model can explain the mechanism of the pathological Internet to a greater extent, and the comparative model is retained.

**Table 3:** Comparison of partial and full mediation models.

	*χ^2^*	*df*	*χ^2^* /*df*	CFI	NNFI	IFI	RMSEA	*∆R^2^*
Theoretical model	22.884	6	3.814	0.996	0.974	0.996	0.049	0.242
Comparison model	20.233	9	2.248	0.997	0.990	0.997	0.033	0.243

*Based on the theoretical model, the comparison model reduces the paths from Big Five personality to PIU and Big Five personality to loneliness to depression*.

From the perspective of the comparative model, the influence of Big Five personality traits on PIU includes 8 paths, combined with the standardized estimation results of variables’ direct and indirect effects (Table 4). The results show that the total effect of Big Five personality traits on PIU is 0.63; the direct effects of extroversion and conscientiousness on the pathological Internet accounted for 14.29 and 47.62% of the total effect, respectively; and the indirect effects of extroversion, agreeableness, and neuroticism on the pathological Internet through loneliness accounted for 2.89, 6.67, and 6.44% of the total effects, respectively. The indirect effects of agreeableness, responsibility, and neuroticism on the pathological Internet through depression accounted for 7.33, 2.67, and 12.33% of the total effect.

**Table 4:** The influence path of the five personalities on PIU.

Path	Standardized path coefficient	Effect size
Extraversion→PIU	0.09	
Conscientiousness→PIU	0.30	
Extraversion→Loneliness→PIU	−0.02	2.89%
Agreeableness→Loneliness→PIU	−0.04	6.67%
Neuroticism→Loneliness→PIU	0.04	6.44%
Agreeableness→Depression→PIU	−0.05	7.33%
Conscientiousness→Depression→PIU	−0.02	2.67%
Neuroticism→Depression→PIU	0.08	12.33%
Total effect	0.63	

## Discussion

### Direct Effect of Big Five Personality Traits on PIU

The Big Five personality traits have an important influence on pathological Internet use. The results of the correlation analysis show that neuroticism is positively correlated with PIU, and extraversion, pleasantness, a sense of responsibility, and openness are significantly negatively correlated with PIU, which is consistent with the results of existing studies (Wang and Zhao, 2000; Kong et al., 2011). The structural equation results show that only extraversion and sense of responsibility have a predictive effect on PIU, among which extraversion has a significant positive predictive effect on PIU, and sense of responsibility has a significant negative predictive effect on PIU. This finding is consistent with the results of existing studies (Yang et al., 2006, 2007). In addition, this study introduced two mediating variables of loneliness and depression, further revealing that individuals with different personality characteristics have different PIU formation mechanisms. The results showed that loneliness and depression can significantly promote PIU.

Specifically, a sense of responsibility is negatively correlated with PIU. Responsible people tend to attach great importance to their own work and academic performance, often give priority to academic and educational goals, are able to plan and have self-control, and have a strong sense of responsibility to their families. This sense of responsibility can help them better resist the negative influence of the Internet and better control their thoughts and behaviors. At the same time, college students with a strong sense of responsibility tend to take their studies more seriously and responsibly and are more likely to succeed in their studies, thus making them more capable of resisting the temptation of the Internet. Conversely, college students with a poor sense of responsibility are prone to adopt negative coping styles when they encounter setbacks, which will lead them to seek success in the virtual world of the Internet, escape reality, seek pleasure, and indulge on the Internet, which leads to PIU (Li and Wang, 2013). Extroversion can positively predict PIU. People with an extroverted personality tend to be inclined toward the outside world in their psychological activities, often show concern and interest in objective things, have a cheerful and lively personality, are willing to participate in group activities, like a lively environment, and are good at socializing. However, they are unwilling to contemplate and often ask for help from others to meet their emotional needs. Such people tend to be impulsive and tend to seek new stimuli. There are endless new things on the Internet, which will bring them new stimulations. Therefore, people with extroverted personalities are more likely to indulge on the Internet (Zhou et al., 2017).

### Mediating Role of Loneliness

This study found that neuroticism, agreeableness, and extraversion in the Big Five personality traits can influence pathological Internet use through the mediating effect of loneliness. These findings support personality mediation result theory (Zamani et al., 2011); that is, personality affects individual behavior in a variety of ways, and the influence of personality on outcomes can occur through mediation. According to personality mediation result theory, loneliness is an important mediator in the relationship between personality and PIU; that is, individuals with different personality characteristics can influence PIU through loneliness. This research conclusion is consistent with previous research (Carver and Smith, 2010). According to stress-coping theory, when people’s loneliness is too severe and their emotions are out of control, emotional focus coping may lead to undesirable situations, which will further trigger negative emotions. In this case, individuals are more likely to indulge on the Internet to eliminate negative emotions, leading to PIU (Lightsey and Hulsey, 2002).

In addition to the overall analysis results, we also separately examined the differences in the path of action of different personality characteristics in personality-loneliness PIU. First, an exploration of the relationship between Big Five personality traits and loneliness shows that extraversion, neuroticism, and agreeableness can effectively predict loneliness, whereas responsibility and openness have no significant impact on loneliness. Extraversion and agreeableness can negatively predict loneliness. Specifically, extroverted individuals have obvious characteristics of impulsivity. Extraversion includes imagination, creativity, curiosity, flexibility, and the tendency to adapt to the heart, and agreeableness includes the traits of trust, altruism, straightforwardness, compliance, and modesty. Precisely because of the influence of this personality trait do they take the initiative to interact with people, producing loneliness in their hearts and producing PIU are not easy. Neuroticism can positively predict loneliness, which shows that it is based on avoidance temperament and tends to lead to experiences of fear, sadness, and pain. Thus, neuroticism is not good for communicating with people and leads to loneliness because people with strongly neurotic personalities are more talkative on social networks. However, the content that they post is mostly negative because they are more inclined to express their painful inner emotions when they are alone, and social networks only provide them with a way to vent. These findings are consistent with the biological perspective and previous research (Zhang et al., 2013).

### Mediating Role of Depression

The cognitive-behavioral model proposed by Davis (2001) is mainly used to explain the development and maintenance of “pathological Internet use.” According to his explanation, individuals do not experience the negative consequences of using the Internet, but their existing social and psychological problems (such as depression) include various maladjustments (for example, PIU use) caused by Internet use (Kumar et al., 2018). In addition, the mood enhancement hypothesis proposed by Bryant and Zillmann states that when individuals have psychological problems, such as depression and anxiety, they will determine the time and type of Internet use according to their own emotional states. To eliminate negative emotions, such as depression, individuals will spend more time on online chats and online games, which increases the probability of PIU usage behavior (Wang et al., 2017).

This study found that neuroticism, responsibility, and pleasantness can significantly predict the level of depression in an individual. The specific manifestation is that neuroticism can positively predict depression. People with a neurotic personality will experience strong mental anxiety when facing psychological difficulties, which will lead to misunderstandings, uneasiness, and discomfort, in contrast to healthy people. Physical, mental, and physiological changes are mistaken for morbidity or abnormality. Therefore, neuroticism tends to have a negative impact on an individual’s career, love, life, and interpersonal relationships. Individuals with neuroticism tend to experience more difficult relationships and predicaments, which makes them easily depressed and prone to depression when experiencing difficulties. They are more likely to indulge on the Internet to avoid these unpleasant experiences.

Responsibility and agreeableness can negatively predict depression. Responsibility includes the characteristics of, for example, competence, fairness, organization, due diligence, achievement, self-discipline, prudence, and restraint (Long et al., 2021). Responsibility also reflects the degree of individual self-control and the ability to postpone the satisfaction of needs. Responsible people are diligent and purposeful and have a sense of direction in life; therefore, they are less depressed. Because highly agreeable people are more considerate and friendly, willing to give up their own interests for others, are optimistic about human nature, and believe that human nature is inherently good, they have better interpersonal relationships and higher levels of social support; thus, they are highly agreeable and not prone to depression or PIU Liu and Zhang, 2020).

### Research Implications and Deficiencies

Our results have important practical implications. First, people should pay attention to the individual differences of the Big Five personality traits on PIU: individuals with conscientious and agreeableness personality traits were less likely to have PIU, whereas individuals with neuroticism had more PIU. Therefore, when we intervene in individual PIU, we need to consider neurotic individuals as the key intervention targets. Second, our research results can help practitioners understand the mediating role of loneliness and depression in the influence of Big Five personality traits on PIU and suggest possible ways of intervention. For example, psychological counseling, quality development, and other activities to relieve the loneliness and depression of college students help them escape psychological problems and reduce PIU behavior.

At the same time, this study has the following limitations. First, although we have investigated the influence of personality on PIU under the guidance of theory, the cross-sectional research design cannot explore the causal relationship between Big Five personality traits and PIU. Therefore, future research should use tracking research to better explore the mechanism of change between Big Five personality traits and PIU. Second, because the subjects of this study were college students, the research results may not be suitable for promotion to a larger age range or a wider group of subjects. Future research can extend to other age groups, such as elementary school students, middle school students, other social backgrounds, countries, and others, to explore whether the research results are consistent across ages and cultures. Third, the internal consistency reliability analysis, structural validity, discriminant validity, and combination reliability of the scales used in this study are all great, but the AVE is an infrequently reported value, which of the Big Five Personality Scale, depression Scale, and Loneliness scale are low, so scales with better convergence validity will be used in future studies. Finally, this study examines the relationship between Big Five personality traits and PIU. Because PIU also has different subtypes, future research should also examine the relationship between Big Five personality traits and different subtypes of PIU.

## Data Availability Statement

The raw data supporting the conclusions of this article will be made available by the authors, without undue reservation.

## Author Contributions

YZ conducting a survey, data collection and sorting, and writing review and editing. LH is responsible for data collection, review, review and edit, and put forward constructive suggestions for revision. HL and SY investigates, writes, and edits. We participate in the investigation, review, editing, and supervision. All authors contributed to the article and approved the submitted version.

## Funding

This research was supported by the Social Science Planning Project of Jinan City in 2018, Research on the Role of Ideological and Political Education in Online Positive Energy Transmission (JNSK18D24).

## Conflict of Interest

The authors declare that the research was conducted in the absence of any commercial or financial relationships that could be construed as a potential conflict of interest.

## Publisher’s Note

All claims expressed in this article are solely those of the authors and do not necessarily represent those of their affiliated organizations, or those of the publisher, the editors and the reviewers. Any product that may be evaluated in this article, or claim that may be made by its manufacturer, is not guaranteed or endorsed by the publisher.
